# Will Benchtop Sequencers Resolve the Sequencing Trade-off in Plant Genetics?

**DOI:** 10.3389/fpls.2016.00433

**Published:** 2016-04-06

**Authors:** Alex D. Twyford

**Affiliations:** Ashworth Laboratories, Institute of Evolutionary Biology, The University of EdinburghEdinburgh, UK

**Keywords:** genomics, next generation sequencing, benchtop sequencers, Illumina MiniSeq, population genetics, phylogenetics, parentage analysis

An important experimental design consideration in plant genetics is the trade-off between number of individuals and number of loci that can be genotyped (Davey et al., 2011). For any given study, an investigator must choose how they partition research effort and resources, with the generation of many loci usually coming at the expense of many individuals, and *vice versa*. For example, for parentage and paternity analysis it is usually more important to sample many individuals (e.g., Andrew et al., 2013), while for comparative genome evolution the emphasis is firmly placed on recovering more loci (Figure 1). This trade-off still exits despite the plummeting costs of sequence data, with researchers having to decide the number of individuals feasible for a given sequencing strategy, and how the libraries will be multiplexed across lanes of a next generation sequencing (NGS) platform (Shen et al., 2011).

**Figure 1:**
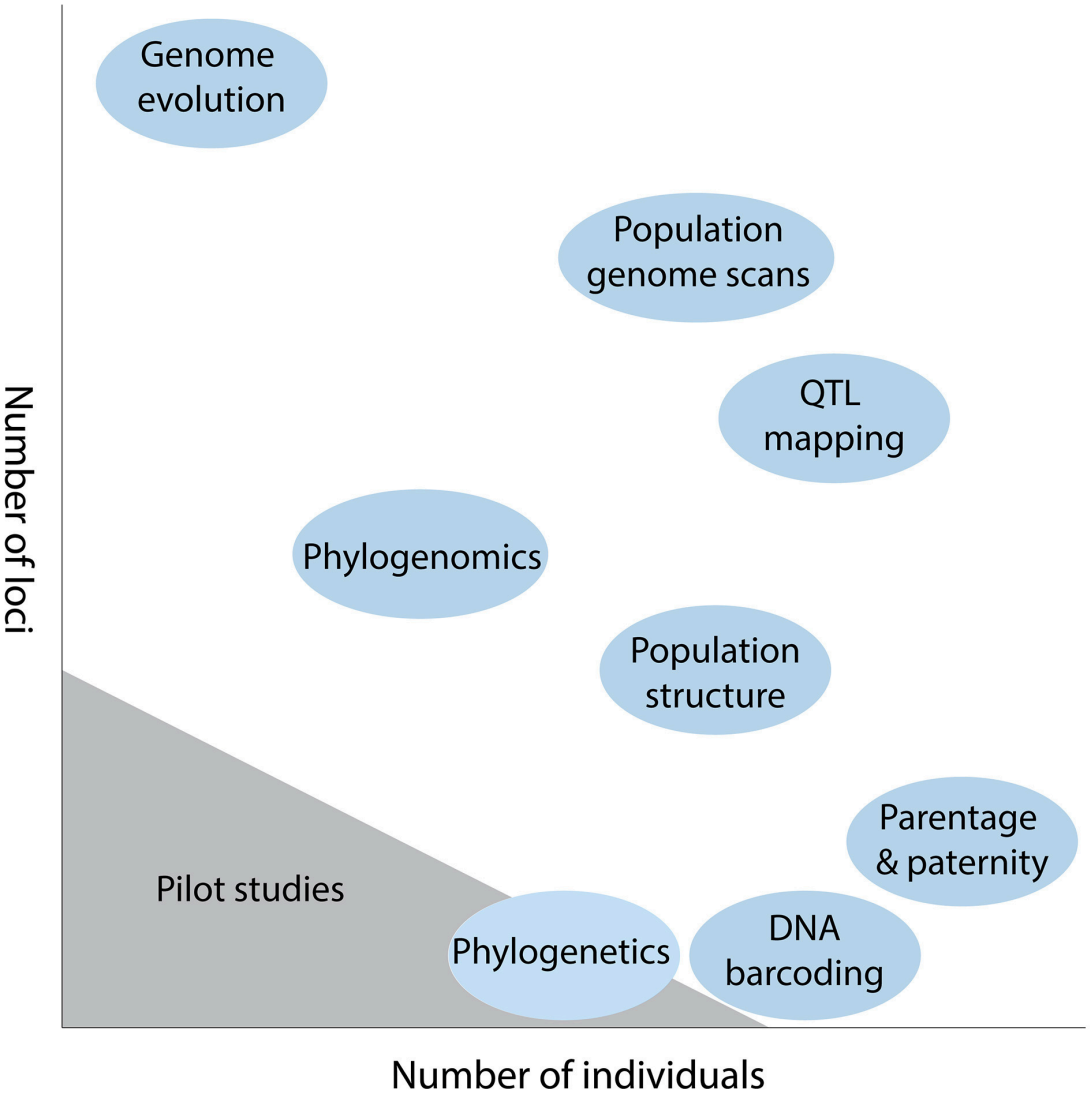
**Diagrammatic representation of the trade-off between number of loci and number of individuals in some typical plant genetic studies**. The scale of sequencing for pilot studies is also indicated.

NGS is well-suited to studies requiring large amounts of sequence data for few individuals, such as *de novo* genome assembly, or large-scale genome resequencing projects (e.g., Brandvain et al., 2014). At the other extreme, high-throughput sequencing is also ideal for single-locus studies of environmental variation, where universal primers are used to amplify a diverse mix of template DNA representing thousands of individuals (e.g., Shokralla et al., 2012). The sequencing trade-off space traditionally least well-served by NGS is where tens or hundreds of loci need to be generated for many individuals. While Restriction-site Associated DNA (RAD) sequencing and genotyping-by-sequencing (GBS, Elshire et al., 2011) partly fill this gap, there are many applications in population genetics, phylogenetics, DNA barcoding, and parentage analysis where a standard multiplexed RAD library run on a high-throughput sequencer would provide an excessive number of loci or unnecessarily high depth-of-coverage. Therefore, researchers wanting a modest number of loci would be more likely to consider either SNP chips, which can be costly to develop and may produce data with ascertainment bias (Albrechtsen et al., 2010), or continue using conventional markers such as microsatellites, or Sanger Sequencing of individual loci.

The uptake of NGS in small to medium-scale studies may be set to increase with the recent announcement of a new benchtop sequencing platform, the Illumina MiniSeq (http://www.illumina.com/miniseq). This sequencer has two key benefits over its larger cousins such as the Illumina HiSeq. Firstly, the MiniSeq fills a gap at the low-read production end of the market, generating 1.8–7.5 Gb of data [8–50 million (M) reads]. These data have a low error rate (>80% bases >Q30), and the platform offers some flexibility over read length configuration [36, 50, 75, 150 bp single end (SE) or paired end (PE) sequences]. Secondly, the MiniSeq is the first Illumina platform designed for smaller research institutions or individual laboratories. The instrument itself costs around $50,000, has a small footprint, relatively short run time, and the capacity to sequence a single sample (rather than the need to fill multiple lanes of a larger flow cell). As such, this sequencer may enable users to avoid queues and administration associated with large sequencing centers, and open up in-house genomics for the first time.

The MiniSeq joins a number of other NGS platforms capable of relatively small sequence runs (e.g., 400 Mb–15 Gb), such as the Illumina MiSeq and ThermoFisher's Ion Torrent, and third generation technologies such as Oxford NanoPore and Pacific BioSciences real-time sequencers (for full comparison see http://www.molecularecologist.com/next-gen-fieldguide-2016/). The MiniSeq's small footprint and low upfront cost make it a more attractive option for lab ownership than the MiSeq, and also boasts the lowest reagent costs for small Illumina sequencing runs (MiniSeq mid-output reagents $550 per run). However, the MiniSeq offers no cost benefits for higher-output runs, and has a shorter maximum read length than the MiSeq (150 bp as opposed to 300 bp). Ion Torrent systems such as the Ion S5 are another low-output benchtop alternative to the MiniSeq, and the fast run time make them the platform of preference for clinical diagnostics. Ion Torrent has not widely been used for non-model genomics (though see Recknagel et al., 2015), likely due to some sequence biases, moderate error rates, and difficulty reading homopolymer regions, particular with early release platforms (Loman et al., 2012; Quail et al., 2012; Salipante et al., 2014). Third generation sequencing options are Oxford NanoPore's MinION (Mikheyev and Tin, 2014) or Pacific BioSciences real-time sequencers (Jiao et al., 2013). While the long sequence reads (>5 Kb) make them extremely useful for *de novo* assembly of small genomes, and scaffolding non-model genomes (English et al., 2012), they have not been widely adopted for other research applications due to their high costs, error rates, and currently limited (but growing) number of bioinformatic pipelines.

The potential applications of low-output benchtop sequencers, such as the MiniSeq, are huge. The first important use would be in replacing panels of PCR-based markers in studies relying on modest numbers of loci. In phylogenetics, multiplexed tagged amplicons could be sequenced with sufficient sequencing depth, but at a cheaper cost and without the redundancy of higher-output platforms. For nuclear loci, this approach removes the time-consuming stage of cloning, and can provide directly phased sequences (O'Neill et al., 2013). Similarly, targeted enrichment studies such as those using hybridization-based probes are ideal for low-output sequencers, as sequencing effort is focused on a small subsection of the genome (e.g., Stull et al., 2013). In mating system studies, GBS libraries prepared with an infrequent cutting enzyme could be a time and cost effective way to generate a modest number of loci in many progeny derived from many seed families, leading to accurate estimates of outcrossing (Koelling et al., 2012). In all these cases, the output of the MiniSeq is optimized for part of the sequencing trade-off where many other platforms are not.

The second main use would be in genomic studies where few individuals need to be sequenced. MiniSeq runs would be suitable for sequencing small plant genomes (e.g., >50X coverage of 135 Mb *Arabidopsis thaliana*), or for characterizing features such as GC-content, transposon composition (Sveinsson et al., 2013), and genome size (Simpson, 2014) of non-model species. This output range could also be useful for multiplexed low coverage genome resequencing (“genome skimming,” Straub et al., 2012), which is proving a popular route for complete plastid assembly (e.g., Jackman et al., 2016). The low sequence run cost would also make this ideal for marker discovery and developing microsatellite primers (Zalapa et al., 2012).

The third use would be for pilot studies testing new sample assays and for validating libraries constructed from difficult samples. Low-output sequencing runs would be extremely valuable to verify the number of tags and the sequencing coverage in test RAD libraries. Similarly, targeted enrichment strategies could be tested at low coverage to check the efficacy of the enrichment and the proportion of off-bait targets. This information can then be used to pick the depth of coverage for large-scale sequencing efforts, with the same Illumina-compatible libraries being transferable across sequencing platforms. For validating samples, low-output sequencing runs could be used to assess the number of informative reads and the extent of sample contamination in dietary or environmental samples (e.g., Willerslev et al., 2014). In studies using degraded herbarium samples, the extent of C → T/G → A miscoding lesions caused by DNA degradation (Staats et al., 2011), could be assessed. This is particularly important as this may not be captured by other quality control metrics, such as those produced by the Agilent TapeStation or Bioanalyser. In all these cases, the small datasets would be able to address issues that would otherwise only come to light with greater sequencing effort.

NGS is providing a number of important solutions to the sequencing trade-off in plant genetic studies, with benchtop sequencers such as the Illumina MiniSeq potentially facilitating day-to-day low-output sequencing. However, the success of such platforms is far from guaranteed. The most cost-effective sequencing comes from high-output platforms such as the Illumina HiSeq 4000, and highly multiplexed libraries or pools of individuals (Pool-seq, Schlötterer et al., 2014) run on such systems have the lowest per-megabase costs. Therefore, current high-output systems may continue to meet most researcher's needs, leaving only a small gap in the market for these platforms. Another issue is the methodological challenges and costs associated with preparing NGS libraries (often $30–100/sample), and the bioinformatics involved in calling reliable variant sites, which may outweigh the benefits of conventional markers for some small-scale studies where these platforms could be useful. A final concern is whether research groups want to own and run their own sequencer, when technical assistance is available at larger sequencing hubs. As such, while the MiniSeq has great potential on paper, whether it really resolves the sequencing trade-off at the low-output end of the market remains to be seen.

## Author contributions

The author confirms being the sole contributor of this work and approved it for publication.

### Conflict of interest statement

The author declares that the research was conducted in the absence of any commercial or financial relationships that could be construed as a potential conflict of interest.

## References

[B1] Albrechtsen A. Nielsen F. C. Nielsen R. (2010). Ascertainment biases in SNP chips affect measures of population divergence. Mol. Biol. Evol. 27, 2534–2547. 10.1093/molbev/msq148 20558595 PMC3107607

[B2] Andrew R. L. Bernatchez L. Bonin A. Buerkle C. A. Carstens B. C. Emerson B. C.. (2013). A road map for molecular ecology. Mol. Ecol. 22, 2605–2626. 10.1111/mec.12319 23611646

[B3] Brandvain Y. Kenney A. M. Flagel L. Coop G. Sweigart A. L. (2014). Speciation and introgression between *Mimulus nasutus* and *Mimulus guttatus*. PLoS Genet. 10:e1004410. 10.1371/journal.pgen.1004410 24967630 PMC4072524

[B4] Davey J. W. Hohenlohe P. A. Etter P. D. Boone J. Q. Catchen J. M. Blaxter M. L. (2011). Genome-wide genetic marker discovery and genotyping using next-generation sequencing. Nat. Rev. Genet. 12, 499–510. 10.1038/nrg3012 21681211

[B5] Elshire R. J. Glaubitz J. C. Sun Q. Poland J. A. Kawamoto K. Buckler E. S.. (2011). A robust, simple Genotyping-by-Sequencing (GBS) approach for high diversity species. PLoS ONE 6:e19379. 10.1371/journal.pone.0019379 21573248 PMC3087801

[B6] English A. C. Richards S. Han Y. Wang M. Vee V. Qu J.. (2012). Mind the gap: upgrading genomes with Pacific Biosciences RS long-read sequencing technology. PLoS ONE 7:e47768. 10.1371/journal.pone.0047768 23185243 PMC3504050

[B7] Jackman S. D. Warren R. L. Gibb E. A. Vandervalk B. P. Mohamadi H. Chu J.. (2016). Organellar genomes of white spruce (*Picea glauca*): assembly and annotation. Genome Biol. Evol. 8, 29–41. 10.1093/gbe/evv244 26645680 PMC4758241

[B8] Jiao X. Zheng X. Ma L. Kutty G. Gogineni E. Sun Q.. (2013). A benchmark study on error assessment and quality control of CCS reads derived from the PacBio, RS.J. Data Mining Genomics Proteomics 4, 16008. 10.4172/2153-0602.1000136 24179701 PMC3811116

[B9] Koelling V. A. Monnahan P. J. Kelly J. K. (2012). A Bayesian method for the joint estimation of outcrossing rate and inbreeding depression. Heredity (Edinb). 109, 393–400. 10.1038/hdy.2012.58 22990309 PMC3499842

[B10] Loman N. J. Misra R. V. Dallman T. J. Constantinidou C. Gharbia S. E. Wain J.. (2012). Performance comparison of benchtop high-throughput sequencing platforms. Nat Biotech. 30, 434–439. 10.1038/nbt.2198 22522955

[B11] Mikheyev A. S. Tin M. M. (2014). A first look at the Oxford Nanopore MinION sequencer. Mol. Ecol. Res. 14, 1097–1102. 10.1111/1755-0998.12324 25187008

[B12] O'Neill E. M. Schwartz R. Bullock C. T. Williams J. S. Shaffer H. B. Aguilar-Miguel X.. (2013). Parallel tagged amplicon sequencing reveals major lineages and phylogenetic structure in the North American tiger salamander (*Ambystoma tigrinum*) species complex. Mol. Ecol. 22, 111–129. 10.1111/mec.12049 23062080

[B13] Quail M. A. Smith M. Coupland P. Otto T. D. Harris S. R. Connor T. R.. (2012). A tale of three next generation sequencing platforms: comparison of Ion Torrent, Pacific Biosciences and Illumina MiSeq sequencers. BMC Genomics 13:341. 10.1186/1471-2164-13-341 22827831 PMC3431227

[B14] Recknagel H. Jacobs A. Herzyk P. Elmer K. R. (2015). Double-digest RAD sequencing using Ion Proton semiconductor platform (ddRADseq-ion) with nonmodel organisms. Mol. Ecol. Res. 15, 1316–1329. 10.1111/1755-0998.12406 25808755

[B15] Salipante S. J. Kawashima T. Rosenthal C. Hoogestraat D. R. Cummings L. A. Sengupta D. J.. (2014). Performance comparison of Illumina and Ion Torrent next-generation sequencing platforms for 16S rRNA-based bacterial community profiling. Appl. Environ. Microbiol. 80, 7583–7591. 10.1128/AEM.02206-14 25261520 PMC4249215

[B16] Schlötterer C. Tobler R. Kofler R. Nolte V. (2014). Sequencing pools of individuals—mining genome-wide polymorphism data without big funding. Nat. Rev. Genet. 15, 49–763. 10.1038/nrg3803 25246196

[B17] Shen Y. Song R. Pe'er I. (2011). Coverage tradeoffs and power estimation in the design of whole-genome sequencing experiments for detecting association. Bioinformatics 27, 1995–1997. 10.1093/bioinformatics/btr305 21636589 PMC3129526

[B18] Shokralla S. Spall J. L. Gibson J. F. Hajibabaei M. (2012). Next-generation sequencing technologies for environmental DNA research. Mol. Ecol. 21, 1794–1805. 10.1111/j.1365-294X.2012.05538.x 22486820

[B19] Simpson J. T. (2014). Exploring genome characteristics and sequence quality without a reference. Bioinformatics 30, 1228–1235. 10.1093/bioinformatics/btu023 24443382 PMC3998141

[B20] Staats M. Cuenca A. Richardson J. E. Vrielink-van Ginkel R. Petersen G. Seberg O.. (2011). DNA damage in plant herbarium tissue. PLoS ONE 6:e28448. 10.1371/journal.pone.0028448 22163018 PMC3230621

[B21] Straub S. C. Parks M. Weitemier K. Fishbein M. Cronn R. C. Liston A. (2012). Navigating the tip of the genomic iceberg: next-generation sequencing for plant systematics. Am. J. Bot. 99, 349–364. 10.3732/ajb.1100335 22174336

[B22] Stull G. W. Moore M. J. Mandala V. S. Douglas N. A. Kates H.-R. Qi X. (2013). A targeted enrichment strategy for massively parallel sequencing of angiosperm plastid genomes. Appl. Plant Sci. 1:1200497. 10.3732/apps.1200497 25202518 PMC4105372

[B23] Sveinsson S. Gill N. Kane N. C. Cronk Q. (2013). Transposon fingerprinting using low coverage whole genome shotgun sequencing in Cacao (*Theobroma cacao* L.) and related species. BMC Genomics 14, 1–12. 10.1186/1471-2164-14-502 23883295 PMC3726317

[B24] Willerslev E. Davison J. Moora M. Zobel M. Coissac E. Edwards M. E.. (2014). Fifty thousand years of Arctic vegetation and megafaunal diet. Nature 506, 47–51. 10.1038/nature12921 24499916

[B25] Zalapa J. E. Cuevas H. Zhu H. Steffan S. Senalik D. Zeldin E.. (2012). Using next-generation sequencing approaches to isolate simple sequence repeat (SSR) loci in the plant sciences. Am. J. Bot. 99, 193–208. 10.3732/ajb.1100394 22186186

